# Hand Functioning in Children with Cerebral Palsy

**DOI:** 10.3389/fneur.2014.00048

**Published:** 2014-04-09

**Authors:** Carlyne Arnould, Yannick Bleyenheuft, Jean-Louis Thonnard

**Affiliations:** ^1^Physical and Occupational Therapy Departments, Paramedical Category, Haute Ecole Louvain en Hainaut, Charleroi, Belgium; ^2^Institute of Neuroscience, Université catholique de Louvain, Brussels, Belgium; ^3^Department of Physical and Rehabilitation Medicine, Cliniques universitaires Saint-Luc, Brussels, Belgium

**Keywords:** cerebral palsy, hand, manual ability, activities of daily living, body functions, dexterity, path analysis, relationships

## Abstract

Brain lesions may disturb hand functioning in children with cerebral palsy (CP), making it difficult or even impossible for them to perform several manual activities. Most conventional treatments for hand dysfunction in CP assume that reducing the hand dysfunctions will improve the capacity to manage activities (i.e., manual ability, MA). The aim of this study was to investigate the directional relationships (direct and indirect pathways) through which hand skills influence MA in children with CP. A total of 136 children with CP (mean age: 10 years; range: 6–16 years; 35 quadriplegics, 24 diplegics, 77 hemiplegics) were assessed. Six hand skills were measured on both hands: touch-pressure detection (Semmes–Weinstein esthesiometer), stereognosis (Manual Form Perception Test), proprioception (passive mobilization of the metacarpophalangeal joints), grip strength (GS) (Jamar dynamometer), gross manual dexterity (GMD) (Box and Block Test), and fine finger dexterity (Purdue Pegboard Test). MA was measured with the ABILHAND-Kids questionnaire. Correlation coefficients were used to determine the linear associations between observed variables. A path analysis of structural equation modeling was applied to test different models of causal relationships among the observed variables. Purely sensory impairments did seem not to play a significant role in the capacity to perform manual activities. According to path analysis, GMD in both hands and stereognosis in the dominant hand were directly related to MA, whereas GS was indirectly related to MA through its relationship with GMD. However, one-third of the variance in MA measures could not be explained by hand skills. It can be concluded that MA is not simply the integration of hand skills in daily activities and should be treated *per se*, supporting activity-based interventions.

## Introduction

Hand functioning, the ability of the hands to perform properly in various contexts, requires the integrity of the central nervous system and, therefore, may be disturbed by different brain disorders. Cerebral palsy (CP) is the most prevalent form of physical disability in children (1), occurring in 1 out of 303 live births (http://www.cdc.gov/ncbddd/cp/index.html). Almost 50% of children with CP present an arm–hand dysfunction (2, 3). Children with unilateral spastic CP seldom use their paretic hand spontaneously in daily activities (2, 4). For these reasons, increasing attention in the last decade has focused on hand functioning in children with CP.

The impact of CP on a child’s hand functioning may be formalized through the theoretical framework of the International Classification of Functioning, Disability, and Health (ICF) (5). According to the ICF, CP may affect three separate but related domains of functioning: body functions and structures (body domain), activities (individual domain), and participation (social domain). In the present work, only the body and individual domains were considered, as the social dimension cannot be reduced to the sole functioning of the hands. Body functions include the physiological or psychological functions of the different body systems. Body structures refer to the anatomic parts of the body (e.g., organs, limbs, and their components). By definition, CP is a consequence of early brain lesions that may affect the corticospinal tract. CP may impact the hand and its components (e.g., muscles, joints, and bones), as well as several body functions (e.g., muscle strength, control of rapid coordinated movements, touch-pressure detection, and recognition of common objects and shapes). CP may also limit the ICF domain of activities, which refers to the ability to execute an essential task or action of daily living (e.g., eating, drinking, grooming, or dressing). In this paper, the term “hand skills” will be used to refer to hand functions (ICF body domain) and hand mobility (ICF activity domain, mobility subdomain). The term “manual ability” (MA) will be used to refer to the children’s capacity to manage daily activities requiring the use of hands and upper limbs (ICF activity domain, self-care subdomain) (6).

One fundamental rehabilitation goal is to improve the child’s ability to manage daily activities necessary for autonomous living (7). Most conventional treatments endeavor to reduce hand impairments by normalizing movement patterns, stretching spastic muscles, strengthening weakened muscles, etc., assuming that body impairments are largely responsible for the difficulties experienced in daily activities (2). However, the ICF stresses the importance of addressing the impact of CP on the child’s hand functioning beyond the body level. The ICF has contributed to a recent shift away from body functions and toward the activities and participation perspectives (8). Recent neurorehabilitation concepts have emphasized what children do in their actual environment, rather than what they can do in a standardized environment (9). Newly developed activity-based interventions, including constraint-induced movement therapy (CIMT) (10) and hand–arm bimanual intensive therapy (HABIT) (11), provide evidence for the improvement of hand functioning (12–14).

Understanding the interrelationships between hand skills and how these are related to MA in children with CP is crucial for planning and implementing the most appropriate rehabilitation interventions. According to previous studies in children and adolescents with CP, MA was not related to passive range of motion (15), but was moderately to highly related to other hand motor skills (e.g., active range of motion, muscle tone/strength/coordination, dexterity, and quality of movement) (3, 15–20). Touch-pressure detection and proprioception were weakly or not associated with MA, whereas two-point discrimination and stereognosis were moderately to highly related to MA (3, 15). However, these studies used correlation coefficients or multiple regression analyses to study the relationships between hand skills and MA. Although informative, these statistical techniques do not account for the potentially complex interrelationships among hand skills, such as causal chains in which some hand skills may influence other mediating variables, which, in turn, may predict the outcome variable (i.e., MA).

Path analysis is a more powerful tool for interpreting the relationships among a set of variables. By including “mediators,” path analysis can identify directional relationships (both direct and indirect pathways) through which hand skills influence MA. To our knowledge, only one study has applied path analysis in children with CP to study the directional relationship among spasticity, weakness, gross motor function, and activities (21). Spasticity and strength had significant indirect effects on activities, through their effects on gross motor function. According to us, gross motor function mediates between body functions and activities as it reflects a combination of both ICF domains. In the same way, dexterity which is one of the hand skills that best predicts MA (3) and the independence in daily activities (22–25) involves both the ICF domains of body functions and activities (i.e., mobility subdomain including lifting/carrying objects, fine hand use, and hand and arm use). Therefore, we hypothesized that dexterity might link hand functions to MA. Our purpose in the present study was to investigate the directional relationships through which hand skills influence MA in children with CP, and to explore whether dexterity mediates the relationships between hand functions and MA.

## Materials and Methods

### Participants

A cross-sectional analysis was conducted with data derived from two existing studies (26, 27) (*n* = 124) and pre-treatment data from an unpublished study investigating the efficacy of intensive bimanual training (*n* = 12). These studies were previously approved by the ethics committee of the Université catholique de Louvain. All children in this study were over 6 years old, to ensure that they had mature manipulative skills in activities of daily living. Children in the study presented no major intellectual deficit (IQ ≥ 60 or normal school level) and were recruited through several centers dedicated to CP. All 12 children from the second (27) and the unpublished studies presented unilateral spastic CP. Consistent with previous hand–arm bimanual intensive trials, children from the unpublished study had to be able to grasp light objects and lift the more affected arm 15 cm above a table surface and were excluded if they presented: (1) uncontrolled seizures, (2) botulinum toxin injections or orthopedic surgery in the upper or lower extremities within the previous 12 months or planned within the study period, and (3) visual problems likely to interfere with treatment/testing. The entire sample included mainly but not exclusively children with spastic CP (84% spastic syndrome, 4% dyskinetic syndrome, 1% ataxic syndrome, and 11% mixed syndrome). The participant characteristics are shown in Table 1.

**Table 1:** **Participants’ characteristics (*n* = 136)**.

Characteristics	*n*
Age (years)	10.0 ± 2.6 (6–16)
**SEX**	
Girls	56
Boys	80
**LIMB DISTRIBUTION**	
Quadriplegia	35
Diplegia	24
Hemiplegia	77
Right	38
Left	39
**SYMPTOMATIC CLASSIFICATION**	
Spastic syndrome	124
Dyskinetic syndrome^a^	5
Ataxic syndrome	2
Mixed syndrome	15
**GMFCS**	
Level I: most independent motor function	61
Level II	38
Level III	12
Level IV	21
Level V: least independent motor function	4

*GMFCS, gross motor function classification system*.

*^a^Athetosic, dystonic, and choreic movements*.

### Outcome measures

Six hand skills were assessed on both hands, starting with the dominant hand (DH): stereognosis (S), proprioception (P), touch-pressure detection (TD), grip strength (GS), gross manual dexterity (GMD), and fine finger dexterity (FFD). Using the modified Manual Form Perception Test, S was determined as the number of objects out of 10 that a child could correctly identify by touch (28). P was measured by passively moving the metacarpophalangeal joints of the thumb and index finger, and counting the number of joint movement directions that a blindfolded child correctly identified out of 10 trials (5 each for the thumb and index finger) (28). TD was measured by applying the filaments of the Semmes–Weinstein esthesiometer (Lafayette Instrument Company, Loughborough, UK) to the tip of a blindfolded child’s index finger, and recording the force required to bend the thinnest filament that the child could detect (29). GS was determined as the average maximal force exerted on a Jamar hydraulic hand dynamometer (Therapeutic Equipment Corporation, Clifton, NJ, USA) across three trials (30). Using the Box and Block Test (31), GMD was determined as the maximum number of blocks transported individually from one compartment of a box to another in 1 min (32). FFD was measured from three trials of the Purdue Pegboard Test (33) (Lafayette Instrument Company, Sagamore Parkway North, USA) as the average number of pegs picked up from a cup and placed into the holes of a board within 30 s (34).

Manual ability was measured with the ABILHAND-Kids questionnaire (26). For each child, the child’s parents rated 21 mostly bimanual activities on a 3-level response scale (0: impossible, 1: difficult, or 2: easy), according to their child’s perceived difficulty in performing the activity. Each activity had to be completed without technical or human assistance, regardless of the limb(s) or adaptive strategies used. Activities not attempted in the last 3 months were not scored and were encoded as missing responses. As reported in a previous study (26), ordinal total scores obtained on the ABILHAND-Kids questionnaire were transformed into interval-level measures according to the Rasch model (35). Interval-level measures were expressed in logits (i.e., the natural logarithm of the odds of success of a child for an activity). These measures were subsequently recalculated into the percentage of the range of logit measures of the scale (0–100), to facilitate their clinical interpretation.

### Data analysis

Descriptive statistics were performed for each variable, to examine the children’s clinical characteristics. Pearson’s correlation coefficients were used to explore the magnitude of bivariate linear associations among hand skills and between hand skills and MA, according to Guilfords’ guidelines (36).

All hand skills that significantly related to MA were subsequently included in a path analysis of structural equation modeling to test a set of multiple regression equations simultaneously, and to assess the directional relationships (both direct and indirect) through which the predictors influence the outcome variable (37).

Path analysis requires the development of one hypothesized initial model (e.g., of the directional relationships among the set of variables) that is tested against the observed data and progressively refined through successive analyses to fit the data. The theoretical initial model was based on evidence from ICF theoretical considerations (5), relevant literature, and bivariate results. The maximum likelihood method was used to estimate the strength and significance of hypothesized connections among the variables included in the path model. Both unstandardized and standardized path coefficients were estimated.

Unstandardized path coefficients indicate the expected amount of change in MA per unit change in one predictor, while all other predictors are controlled. Unstandardized path coefficients cannot give the relative contribution of each predictor to each dependent variable because they reflect the different metrics used to assess the variables. However, they are useful for testing the path model with a different sample, or with the same sample at different time points. Standardized path coefficients indicate the expected amount of change in MA per standard deviation (SD) change in one predictor, while all other predictors are controlled. Standardized path coefficients estimate the magnitude of relationships among different variables. They can be understood as correlation measures showing the direct effect of an independent or mediating variable on a dependent variable when other predictors are controlled. Non-significant path coefficients imply that the parameters do not differ from zero and could be deleted from the model.

Various fit indices were used to assess the adequacy of the hypothesized path model and to determine how well it explains the data (37). A good fit of the model to the data is indicated by a non-significant chi-squared (χ^2^) statistic (*p* > 0.05), a root mean square error of approximation (RMSEA) below 0.06 (with a lower bound of the 90% confidence interval <0.05 and an upper bound <0.10), an adjusted goodness-of-fit index above 0.90, and goodness-of-fit, normed fit, comparative fit, and Tucker–Lewis indices above 0.95 (37, 38).

Path analysis also provides modification indices, which suggest causal pathways that may be added to improve the goodness-of-fit indices. Additional pathways were only included in the model if they made sense clinically. The path model was modified several times by systematically removing non-significant path coefficients and adding the causal pathways suggested by the modification indices, until the goodness-of-fit indices indicated that the path model fit the data well. Predictive fit indices favoring simpler models, including the Akaike information criterion (AIC), consistent Akaike information criterion (CAIC), and Bayes information criterion (BIC), were considered to choose the more parsimonious model (37). The path model with the lowest AIC, CAIC, and BIC values was chosen as the final model.

The Statistical Package for Social Sciences (SPSS) version 20.0 was used for all statistical analyses. AMOS version 21.0 was used for the path analysis. All assumptions underlying the path analysis were verified; namely, the linearity, normality, and constant variance of the residuals, the absence of influential outliers, and the absence of multicollinearity. To prevent problems with collinearity, when independent variables were intercorrelated by more than 0.80, only one variable was selected. Selection was made on the basis of the clinical sense and the magnitude of the relationship with the dependent variable. The alpha level of significance was fixed at 0.05 for all statistical tests.

## Results

### Descriptive analysis of hand skills and manual ability

Table 2 summarizes the measures of hand skills and MA. Raw scores for hand motor skills were converted into standardized scores (*Z*-scores), according to normative data (30, 34, 39). For our sample, the mean MA measure was 63 ± 22 on a logit scale from 0 to 100. All hand skills were more impaired in the non-dominant hand (NDH) compared to the DH. Gross motor and FFD deficits were observed in both hands for all CP types. This finding indicates that in hemiplegics, the dexterity of the “non-paretic” hand may also be affected, especially in the achievement of fine finger movements. Children with CP were more severely affected in their dexterity compared to other hand skills.

**Table 2:** **Descriptive statistics of manual ability and hand skills**.

Variables	Mean	SD	Median	Q1	Q3	Range	*Z*-score (mean ± SD)
**DEPENDENT VARIABLE**							
Manual ability (% logits)	62.69	22.00	–	–	–	0–100	–
**INDEPENDENT VARIABLES: HAND SKILLS**							
S_DH (n/10)	–	–	10.00	9.00	10.00	1–10	–
S_NDH (n/10)	–	–	9.00	6.00	10.00	0–10	–
P_DH (n/10)	–	–	10.00	10.00	10.00	0–10	–
P_NDH (n/10)	–	–	10.00	8.25	10.00	0–10	–
TD_DH [log_10_ (10 × mg)]	–	–	2.80	2.40	3.20	2–7	–
TD_NDH [log_10_ (10 × mg)]	–	–	2.80	2.40	3.60	2–7	–
GS_DH (kg)	13.77	7.77	–	–	–	0–42	−1.93 ± 1.67
GS_NDH (kg)	7.37	6.17	–	–	–	0–27	−3.06 ± 1.64
GMD_DH (n/1 min)	39.61	18.28	–	–	–	0–86	−2.40 ± 2.72
GMD_NDH (n/1 min)	24.33	16.82	–	–	–	0–67	−4.88 ± 2.80
FFD_DH (n/30 s)	8.69	4.70	–	–	–	0–18	−5.38 ± 4.16
FFD_NDH (n/30 s)	3.14	4.01	–	–	–	0–14	−7.78 ± 3.24

*SD, standard deviation; Q1, first quartile; Q3, third quartile; DH, dominant hand; NDH, non-dominant hand; *n*, number; S, stereognosis; P, proprioception; TD, touch-pressure detection; GS, grip strength; GMD, gross manual dexterity; FFD, fine finger dexterity*.

### Bivariate associations within hand skills and with manual ability

Table 3 reports the correlation coefficients among hand skills and between hand skills and MA. In both hands, MA was significantly but moderately related to hand motor skills and S, but weakly related to P. Although MA was not significantly related to TD in the DH, it was weakly related to TD in the NDH. GMD and FFD presented the highest correlations with MA for both hands, followed by S in the DH and GS in the NDH. In both hands, GMD and FFD were very highly intercorrelated (≥0.87).

**Table 3:** **Pearson correlation coefficient matrix of hand skills and manual ability**.

Variables	1	2	3	4	5	6	7	8	9	10	11	12	13
1. Manual ability (% logits)	1.00												
2. S_DH (n/10)	0.61***	1.00											
3. S_NDH (n/10)	0.42***	0.37***	1.00										
4. P_DH (n/10)	0.31***	0.58***	0.35***	1.00									
5. P_NDH (n/10)	0.25**	0.25**	0.53***	0.57***	1.00								
6. TD_DH_log [log_10_ (10 × mg)]	−0.12	−0.30***	−0.09	−0.41***	−0.22*	1.00							
7. TD_NDH_log [log_10_ (10 × mg)]	−0.21*	−0.27**	−0.59***	−0.41***	−0.55***	0.57***	1.00						
8. GS_DH (kg)	0.48***	0.45***	0.11	0.14	0.02	−0.15	−0.09	1.00					
9. GS_NDH (kg)	0.57***	0.32***	0.54***	0.17*	0.35***	−0.05	−0.30***	0.54***	1.00				
10. GMD_DH (n/1 min)	0.69***	0.55***	0.17*	0.24**	0.09	−0.29***	−0.20*	0.69***	0.43***	1.00			
11. GMD_NDH (n/1 min)	0.72***	0.43***	0.59***	0.23**	0.36***	−0.12	−0.37***	0.36***	0.76***	0.61***	1.00		
12. FFD_DH (n/30 s)	0.68***	0.57***	0.09	0.24**	−0.01	−0.28***	−0.14	0.63***	0.29***	0.90***	0.53***	1.00	
13. FFD_NDH (n/30 s)	0.57***	0.28**	0.52***	0.15	0.34***	−0.09	−0.32***	0.15	0.68***	0.43***	0.87***	0.39***	1.00

*DH, dominant hand; NDH, non-dominant hand; S, stereognosis; P, proprioception; TD, touch-pressure detection; GS, grip strength; GMD, gross manual dexterity; FFD, fine finger dexterity*.

*p < 0.05; **p < 0.01; ***p < 0.001.

To prevent problems with collinearity, only GMD was selected for path analyses. A high association was observed between GMD and GS in the NDH. In both hands, S was moderately related to all other motor and sensory hand skills, except TD in the DH, for which a weak relationship with S was observed. Overall, moderate relationships were found among sensory hand skills. Weak relationships appeared between sensory (TD, P) and motor hand skills, except for GS in the DH, which was only related to S. Moderate to high correlations were observed among hand motor skills. Finally, weak (S and FFD) to moderate (P, TD, GS, and GMD) associations were found between hands for each hand skill.

### Path model of hand functioning in children with cerebral palsy

**Figure 1:**
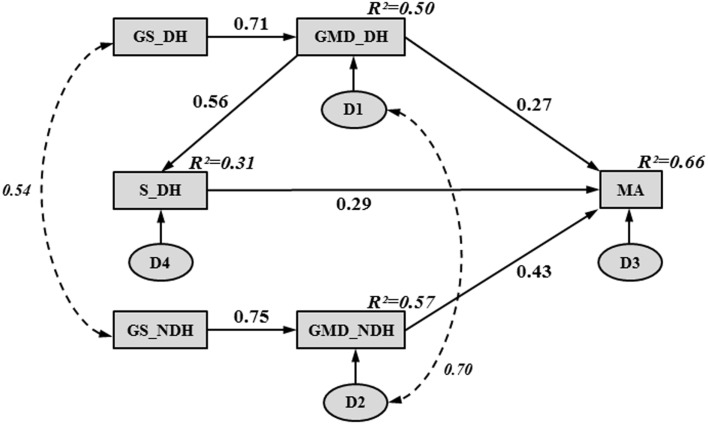
**Final path model, illustrating hand functioning in children with CP**. Rectangles and ovals represent observed and unobserved variables, respectively. A single-headed arrow indicates a direct effect between two variables, pointing from the “cause” (arrow tail) to the “effect” (arrow head). A curved, double dashed arrow indicates a correlation between two variables without any causal assumption. Numbers beside the single- and double-headed arrows correspond to standardized path coefficients. Numbers in the upper right-hand corner of each rectangle represent squared multiple correlations (*R*^2^) (i.e., proportion of the variance in the dependent variable accounted for by the set of independent variables). The letter “D” inside an oval represents the unobservable disturbance (i.e., measurement error and the variance amount of a dependent variable unexplained by the predictors) associated with each dependent and mediating variable.

Figure 1 illustrates the final path model of hand functioning in children with CP. The entire model accounted for 66% of the variance in MA. The chi-squared value (χ^2^ = 8.12, 7 df, *p* = 0.32) representing the overall goodness-of-fit was not significant, supporting the fit of the path model. The path model showed an adequate fit to the data according to all of the other fit indices, except for the upper bound of the 90% confidence interval of the RMSEA (i.e., 0.12), which was slightly higher than the optimal fit criterion (i.e., <0.10).

Table 4 reports the unstandardized path coefficients, their associated standard error (SE), and their significance. All causal pathways were significant, demonstrating that all parameter estimates differed significantly from zero. Table 4 also shows the standardized path coefficients (β; see Figure 1), which reflect the relative importance of each causal pathway. The GMD in both hands (β_GMD_DH→MA_ = 0.27; β_GMD_NDH→MA_ = 0.43; *p* < 0.001) and S in the DH (β_S_DH→MA_ = 0.29; *p* < 0.001) were the only hand skills to contribute directly to MA. The GS and purely sensory skills (P, TD) did not have a significant direct relationship with MA. However, in both hands, GS indirectly contributed to MA through its impact on GMD (β_GS_DH→GMD_DH_ = 0.71; β_GS_NDH→GMD_NDH_ = 0.75; *p* < 0.001). The GS in the DH and NDH explained 50 and 57% of the variance in GMD, respectively. The GMD in the DH was indirectly related to MA through its influence on S (β_GMD_DH→S_DH_ = 0.56; *p* < 0.001) and accounted for 31% of the variance in S.

**Table 4:** **Maximum likelihood parameter estimates for the final path model of hand functioning in children with CP**.

Parameter	Unstandardized	SE	*p*-Value	Standardized
**DIRECT EFFECTS**				
GS_DH → GMD_DH	1.71	0.11	<0.001	0.71
GS_NDH → GMD_NDH	2.01	0.12	<0.001	0.75
GMD_DH → S_DH	0.04	0.01	<0.001	0.56
GMD_DH → MA	0.32	0.08	<0.001	0.27
GMD_NDH → MA	0.56	0.08	<0.001	0.43
S_DH → MA	4.30	0.91	<0.001	0.29
**COVARIANCES**				
GS_DH ↔ GS_NDH	25.61	4.65	<0.001	0.54
D1 ↔ D2	99.20	14.94	<0.001	0.70
**VARIANCES**				
GS_DH	59.88	7.29	<0.001	
GS_NDH	37.85	4.61	<0.001	
D1 (GMD_DH)	172.63	21.04	<0.001	
D2 (GMD_NDH)	117.31	14.29	<0.001	
D3 (MA)	160.69	19.56	<0.001	
D4 (S_DH)	1.47	0.18	<0.001	

*SE, standard error; DH, dominant hand; NDH, non-dominant hand; GS, grip strength; GMD, gross manual dexterity; S, stereognosis; MA, manual ability; D, disturbance (i.e., unexplained variance)*.

Table 5 shows the standardized direct, indirect, and total contributions of hand skills on MA. The indirect effect was calculated as the product of the direct effects that comprised it. For instance, in the DH, GS indirectly affected MA through two pathways: its direct influence on GMD (0.71 × 0.27 = 0.19) and its indirect influence on S (0.71 × 0.56 × 0.29 = 0.12). Thus, the global indirect effect of GS in the DH on MA was equal to 0.31. A similar indirect contribution of GS on MA was observed in the NDH (0.75 × 0.43 = 0.32). Among all of the hand skills investigated in the study, GMD were the strongest contributors to MA in both hands. Although GMD in the NDH had a higher direct impact on MA (β_GMD_NDH→MA_ = 0.43) than GMD in the DH (β_GMD_DH→MA_ = 0.27), similar total contributions were found for both hands due to the indirect effects of GMD on MA through S in the DH (β_GMD_DH→S_DH→MA_ = 0.16).

**Table 5:** **Direct, indirect, and total effects of hand skills on manual ability**.

Hand skills	Effects^a^		
	Direct	Indirect	Total
S_DH	0.29	–	0.29
GS_DH	–	0.31	0.31
GS_NDH	–	0.32	0.32
GMD_DH	0.27	0.16	0.43
GMD_NDH	0.43	–	0.43

*^a^Standardized path coefficients*.

*DH, dominant hand; NDH, non-dominant hand; S, stereognosis; GS, grip strength; GMD, gross manual dexterity; MA, manual ability*.

## Discussion

This study is the first attempt to establish a model for understanding hand functioning in children with CP. According to the path analysis, GMD in both hands and S in the DH were directly related to MA, whereas GS was indirectly related to MA through its relationship with GMD. However, one-third of the variance in MA was not explained by the hand skills investigated in this study.

The path analysis provided a comprehensive picture of hand functioning in children with CP by identifying several mediators through which hand skills influence MA. Among the hand skills investigated, GMD measures in both hands were the strongest contributors to MA. Although related, dexterity is a separate concept from MA. Dexterity refers to the physiological functions of the hand and central nervous system that enable the execution of rapid and coordinated hand movements and mobility, without purposeful functioning. Dexterity tasks are generally performed in a short period of time. Such tasks are not representative of daily activities performed continuously throughout the day, in which fatigue may play a role (39, 40). Moreover, dexterity tasks are too artificial in nature and require too limited of movement patterns to reproduce the meaningful situations encountered in daily life (41, 42). By contrast, MA refers to the use of combined hand functions aimed at executing activities generally considered to be essential for an individual’s daily living. Several factors (e.g., learned non-use phenomenon, motivation, cognitive skills, familial and social environments, etc.) may explain why people with similar dexterity skills might present varying MA levels (3, 40). To prevent problems with collinearity, in this study, only GMD was selected in the path model. GMD was preferred to FFD, as GMD measures in both hands presented the highest correlations with MA. Moreover, in our experience, the Box and Block Test is friendlier and more sensitive than the Purdue Pegboard Test to differentiate more affected CP children. However, a similar path model fitting the data was found when FFD was included instead of GMD.

Apart from GMD in both hands, S in the DH was the only hand skill investigated in the study that contributed directly to MA. A high relationship between S and MA was also previously reported in children with unilateral congenital CP (15). The influence of GMD on S confirms that the recognition of an object by tactile sensation requires that the object be moved in the hand to perceive its shape. Active in-hand manipulation is considered to be more efficient in object identification than passive manipulation (43). Thus, failure to identify some objects by touch might result from manipulative deficits, rather than from real sensory impairments (3). Carlson and Brooks (44) showed that healthy individuals presented reduced S when placed in a simulated hemiplegic hand position compared to a normal hand position. Other studies have confirmed the importance of hand mobility in object recognition, through the moderate associations between S and dexterity (45–49). Our path analysis revealed that GMD could account for 31% of the variance in S.

In both hands, GS indirectly contributed to MA through its impact on GMD, confirming the relationships observed in the literature between hand strength and dexterity (3, 46, 47, 50). Although deficient GS may influence a child’s ability to hold and maintain the grip of objects, objects can be efficiently stabilized in other ways (e.g., against a table surface or body) to perform manual activities. According to Sakzewski et al. (47), a GS >1 kg may be adequate for the NDH to be an effective assisting hand in bimanual tasks. In our sample, only 13% of the children presented a GS below 1 kg in their NDH, and no more than two children were severely affected in both hands. Although children with CP can develop functional compensatory strategies using the less affected hand solely, the path model emphasizes that the success of manual activities, in terms of strength and dexterity, requires cooperation of both hands.

Sensory inputs are important in anticipatory control and grip-lift tasks (43, 51). However, in this study, TD and P were weakly or not related to MA, consistent with other findings in the literature (3, 14, 45). It can be hypothesized that TD and P were not sufficiently impaired in our sample to affect the achievement of manual activities in a significant way (52, 53). Krumlinde-Sundholm and Eliasson (45) and Gordon and Duff (46) found that TD was less impaired than two-point discrimination in children with unilateral spastic CP. They suggested that the children might have had deficient lateral inhibition or tactile spatial resolution, which are required for two-point discrimination (45, 46). The children’s peripheral nerve fibers may have been relatively intact, as reflected by TD (54). TD and P involve low-level sensory processing of somatic stimuli (55). However, higher-level mental body representations (MBRs) that are generated from multisensory inputs are crucial for our daily interaction with the outside environment and may play a role in controlling motor behavior (56). MBRs refer to abstract representations of one’s body derived from sensory inputs, like TD or P, but capable to reciprocally influence primary tactile processing and to modulate the perception of external objects that may be body-referenced, thereby playing a role in perception and/or action (57). MBRs develop slowly during ontogenesis in healthy children (58) and do present abnormalities in cortical activation in children with CP (59, 60). This suggests that children with CP may be unable to fully integrate external stimuli into high-level sensorimotor processes (such as MBRs), which may disturb motor output. Taking MBRs into account in future work [see Gandevia et al. (61) and Longo et al. (62) for MBRs measurement] may reveal whether this aspect contributes to MA.

Disturbances representing the unexplained variances of MA and other mediating variables (GMD in both hands and S in the DH) had to be added in the path model (see Figure 1) since the model is supposed to show all variables that affect the dependent variables. Without disturbances, the path model would make the implausible claim that a dependent variable is measured without any measurement error and is an exact linear combination of the predictors. The significant disturbance covariance between GMD in the DH and NDH indicates that these mediating variables shared at least one common omitted cause (e.g., severity of the disorder). This illustrates the complexity of understanding the relationships among hand skills and between them and MA. Moreover, good fit of a path model does not guarantee that all relevant predictors have been included in the model. Hand skills other than those measured in this work may also impact the achievement of manual activities in CP children. It would be interesting to test the potential contribution on the model of body structures, such as the corticospinal tract dysgenesis measured by the diffusion tensor imaging symmetry index, as a moderate association of this structure with the ABILHAND-Kids questionnaire has been observed (27). Tactile spatial resolution as measured by the grating orientation task should be investigated in the future; unlike the two-point discrimination test, the stimulus-induced neural image is issued only from spatial cues (63). Spasticity is another hand skill that would be interesting to explore in CP children. Reduction of spasticity remains a primary focus in the clinical management of CP, with the assumption that it will lead to an improvement in MA. The reduction of muscle tone (e.g., by botulinum toxin) improves the active range of motion (AROM) of the antagonist muscles, which could create new potentials to learn and improve manual skills (64, 65). However, this was not confirmed by the study of Rameckers et al. (65) showing that though reduced tone leads to an increase in AROM, this gain was not translated into more upper limb function and thus children were not able to benefit from the changes induced by botulinum toxin. Only one study (15) has demonstrated moderate relationships between ABILHAND-Kids measures and spasticity as tested by the Modified Ashworth Scale, a scale that does not comply with the concept of spasticity (i.e., a velocity-dependent increase in muscle tone) (66). Apart from dexterity, other potential mediators between hand functions and MA could be tested in a path model. For instance, the quality of movement, as measured by the Quality of Upper Extremity Skills Test (67) or the Melbourne Assessment of Unilateral Upper Limb Function (68), and the actual use of the affected hand in bimanual activities, as measured by the Assisting Hand Assessment (69), could link hand impairments to MA because they include items related to both the ICF domains of the body functions and activities (i.e., subdomain of mobility) (9).

By exploring the process by which hand skills are related to MA, this study highlights potential treatment priorities to improve hand functioning in children with CP. First, the strengthening of hand muscles may indirectly contribute to improve MA, through its impact on dexterity. In the past, muscle strengthening was not recommended for children with CP because it was believed that muscle strengthening would increase spasticity (21). However, in their study of nine hemiplegic children, Vaz et al. (70) observed significant strength gains due to wrist muscle strengthening by electrostimulation, but no change in passive stiffness. A recent study of 10 children with CP found good short-term efficacy of repetitive intensive strengthening training of the hand, in terms of muscle strength, muscle size, kinematics, and motor function (71). Although additional studies are required to confirm the efficacy of strength training on MA, we believe that weakness of the hand muscles, including spastic muscles, should be treated. Second, dexterity training of children with CP can be helpful to improve MA. The GMD in both hands were the strongest contributors to MA. The GMD mediated the relationship between hand functions and MA, possibly because GMD reflects a combination of both the ICF domains of body functions and activities. Third, our finding that hand skills only partially predicted MA in children with CP has several clinical implications. A therapist cannot assume that an improvement in hand skills will result in a correspondingly higher MA. Interventions focused solely on reducing hand impairments may be questionable, especially as it is more important for CP children to manage daily activities autonomously than it is for them to have “normal” hand functions (72). This conclusion does not mean that interventions based on body functions are useless; indeed, they may be important, especially for preventing secondary impairments (e.g., contractures or deformities) (73). However, as MA is not simply the integration of hand skills in daily activities, MA should be treated *per se*, supporting the usefulness of activity-based interventions such as CIMT or HABIT.

Some limitations should be noted in the interpretations of our findings. The current cross-sectional dataset limits our ability to make causal inferences. A longitudinal study design with multiple end-point measurements is required to ascertain the temporal sequence and to confirm the causal relationships between variables. An additional limitation is the sample size. Although we used a moderate sample size for SEM (37), the necessary number of subjects depends on the model complexity; more parsimonious models (i.e., with less parameter estimates) require smaller samples than more complex models (37). A common sample size guideline for path analysis suggests that 10–20 subjects per parameter are sufficient for reliable model precision (37). Our sample size was adequate, as the ratio of subjects to parameters was 10:1 (i.e., 136/14). As 52% of our sample was constituted by spastic hemiplegic children, the identified path model might reflect the hand functioning of spastic hemiplegics more than that of all CP types. However, children were recruited from different settings (e.g., a CP reference center, university hospitals, special education schools, and rehabilitation centers); thus, the original sampling likely provided a fairly representative CP sample.

The proposed path model is only one possible model of hand functioning in children with CP. A good fit indicates that the model is consistent with the relationships observed in the data. However, there may be other models that also fit the data well. Whenever possible, the relative fit of alternative theoretically plausible models should be considered (74). Several alternative models that included small modifications were tested, all of which presented worse goodness-of-fit and predictive fit indices than the proposed model. Although this result strengthens our confidence in the proposed model, future studies are required to validate our model by confirming models that are based on other independent samples with larger sample sizes and longitudinal study designs (74). The robustness of the model could be tested by selecting other measures of the involved variables. As more evidence is accumulated across studies, we can be more confident in the accuracy of the proposed model.

Although the present study should be regarded as preliminary in light of its limitations, it offers potentially helpful clinical guidelines about the relevant hand skills that should be accounted for when designing hand-care interventions, as well as treatment priorities that should be set up to improve hand functioning in children with CP. Hand muscle strengthening and dexterity training may be useful to improve MA in children with CP. However, MA is not simply the integration of hand skills in daily activities and should be treated *per se*, supporting the usefulness of activity-based interventions.

## Author Contributions

Carlyne Arnould performed the statistical analyses, conducted the literature search, and drafted the manuscript. Carlyne Arnould and Yannick Bleyenheuft participated in the data collection. All authors contributed to the study design, participated in the data interpretation, critically revised the draft of the manuscript for important intellectual content, and contributed to the writing. All authors have read and approved the final manuscript. Data sharing statement: dataset is available from Carlyne Arnould at carlyne.arnould@gmail.com.

## Conflict of Interest Statement

The authors declare that the research was conducted in the absence of any commercial or financial relationships that could be construed as a potential conflict of interest.

## Abbreviations

AIC, Akaike information criterion; AROM, active range of motion; β, standardized path coefficient; BIC, Bayes information criterion; χ^2^, chi-squared statistic; CAIC, consistent Akaike information criterion; CIMT, constraint-induced movement therapy; CP, cerebral palsy; DH, dominant hand; FFD, fine finger dexterity; GMD, gross manual dexterity; GMFCS, gross motor function classification system; GS, grip strength; HABIT, hand–arm bimanual intensive training; ICF, international classification of functioning, disability, and health; IQ, intellectual quotient; MA, manual ability; MBRs, mental body representations; *n*, number; NDH, non-dominant hand; P, proprioception; Q1, first quartile; Q3, third quartile; RMSEA, root mean square error of approximation; S, stereognosis; SD, standard deviation; SE, standard error; TD, touch-pressure detection.
